# Aerobic anoxygenic phototrophs are widespread and active members of Greenland Ice Sheet microbiomes

**DOI:** 10.1093/femsec/fiag116

**Published:** 2026-10-15

**Authors:** Christoph Keuschnig, Helen K Feord, Emily L M Broadwell, Athanasios Zervas, Martyn Tranter, Alexandre M Anesio, Liane G Benning

**Affiliations:** Department Geochemistry, Interface Geochemistry, GFZ Helmholtz Centre for Geosciences, 14473, Potsdam, Germany; Department Geochemistry, Interface Geochemistry, GFZ Helmholtz Centre for Geosciences, 14473, Potsdam, Germany; Department of Environmental Science, Aarhus University, 4000, Roskilde, Denmark; Department of Environmental Science, Aarhus University, 4000, Roskilde, Denmark; Department of Environmental Science, Aarhus University, 4000, Roskilde, Denmark; Department of Environmental Science, Aarhus University, 4000, Roskilde, Denmark; Department Geochemistry, Interface Geochemistry, GFZ Helmholtz Centre for Geosciences, 14473, Potsdam, Germany; Department of Earth Sciences, Freie Universität Berlin, 12249, Berlin, Germany

**Keywords:** supraglacial microbiome, aerobic anoxygenic phototrophs, Greenland Ice Sheet, thiosulfate oxidation, CO oxidation, sulfoquinovose degradation

## Abstract

The Greenland Ice Sheet (GrIS) hosts diverse microbial communities where glacier ice algae, snow algae, and cyanobacteria act as key primary producers across bare ice, snow, and cryoconite habitats, yet the ecological roles of associated heterotrophic prokaryotes remain poorly understood. Aerobic anoxygenic phototrophs (AAPs) are known to couple light harvesting to heterotrophy and enter trophic relationships with algae in marine systems, suggesting they may represent an important but underexplored functional group in supraglacial ecosystems. Using genome-resolved analyses of supraglacial metagenomes and metatranscriptomes from Greenland, we recovered nine AAP metagenome-assembled genomes (MAGs) (4% of the 229 recovered MAGs) consistently enriched in bare ice and cryoconite communities, but nearly absent in snow. A meta-analysis of 2 451 public metagenomes supported this cryospheric habitat association. AAP_MAGs were disproportionally enriched in thiosulfate oxidation and bicarbonate-dependent carbon assimilation pathways relative to other MAGs. Iron and CO oxidation pathways were broadly distributed across the GrIS microbiome, including in AAPs, together suggesting adaptation strategies in supraglacial ecosystems. Genomic evidence for algal coupling via sulfoquinovose degradation was limited. Overall, we document that AAPs are active and widespread in weathering crust habitats, highlighting their potential contributions to biogeochemical cycling in supraglacial ecosystems.

## Teaser

Sunlight-powered bacteria thrive on Greenland's ice, using sulfur and alternative energy sources to survive in the weathering crust where ice melts.

## Introduction

The seasonally melting surfaces of glaciers and ice sheets worldwide host dynamic microbial ecosystems that thrive despite extreme environmental constraints, including low temperatures, high irradiance, and limited nutrient availability (Boetius et al. 2015, Anesio et al. 2017). During the summer melt season, these supraglacial habitats support extensive blooms of pigmented snow and glacier ice algae, which drive local primary production on snow and bare ice surfaces (Lutz et al. 2016, 2018, Williamson et al. 2020, Rossel et al. 2025). This biological darkening reduces surface albedo, accelerates melt rates, and affects ice sheet mass balance (Cook Joseph M. et al. 2020, Chevrollier et al. 2023). While phototrophic primary production on ice and snow surfaces is well established, the ecological roles of associated prokaryotic communities on the Greenland Ice Sheet (GrIS), particularly beyond cyanobacteria in cryoconites (Edwards et al. 2011, Bagshaw Elizabeth A. et al. 2016, Poniecka et al. 2018), remain underexplored.

Supraglacial habitats differ substantially in persistence and, consequently, in their potential capacity to support heterotrophic communities. Seasonal snowpacks are comparatively short-lived, whereas bare ice hosts more persistent microhabitats. These can include cryoconite holes (dark mineral-organic aggregates that absorb solar radiation and melt into the ice surface forming melt-hole habitats) and dispersed cryoconite embedded in the weathering crust, which can recur year after year and remain near the ice surface during ablation (Takeuchi et al. 2018, Irvine-Fynn et al. 2021, Stevens et al. 2022). Importantly, microbes can also overwinter on bare ice: pigmented glacier ice algae, for instance, survive within near surface ice and re-emerge in subsequent melt seasons (Remias et al. 2009). Together, recurring cryoconite rich microhabitats and the persistence of algal cells over the polar winter are expected to support more stable heterotrophic communities and to shape the initial microbial and optical conditions that drive early season surface darkening.

Trophic interactions between algae and heterotrophic bacteria are well documented in non-cryospheric phototrophic ecosystems (e.g. Cirri and Pohnert 2019). In these systems, exudates from phototrophic organisms serve as substrates for heterotrophic communities, which in turn elevate micronutrient availability and provide essential compounds, such as vitamins, to algae (Croft et al. 2005, Seymour et al. 2017).

Aerobic anoxygenic phototrophs (AAPs) were recently documented as abundant and tightly coupled photo-heterotrophs to marine algal blooms (Buchan et al. 2014, Liu et al. 2023), with specific compounds mediating these trophic relationships. Among these, AAPs of the *Roseobacteraceae* family metabolize algal-derived dimethylsulfoniopropionate (DMSP) to dimethyl sulfide, impacting the sulfur cycle in ocean surface waters, atmospheric chemistry, and climate (Wagner-Döbler and Biebl 2006, Ksionzek et al. 2016). Members of the same family were found to degrade sulfoquinovose (SQ), a sulfosugar derived from thylakoid membrane lipids [sulfoquinovosyl diacylglycerol (SQDG)]. SQDG is ubiquitous in chloroplasts of all phototrophs, including eukaryotic algae and cyanobacteria, with an estimated global production of the order of 10 billion tons per year (Harwood and Nicholls 1979, Goddard-Borger and Williams 2017, Nakajima et al. 2018, Snow et al. 2021, Liu et al. 2023, Ma et al. 2025). A similar dynamic between AAPs and algae may therefore also occur in algal-dominated supraglacial systems.

AAPs are a metabolically versatile group of bacteria widely distributed in aquatic ecosystems including oceans, freshwater lakes, and rivers. They combine heterotrophy with light-driven energy generation through bacteriochlorophyll-based anoxygenic phototrophy (Yurkov V and Csotonyi 2009, Ferrera et al. 2017). This combination of traits was shown to offer a competitive advantage in oligotrophic environments (Koblížek 2015). Another light-associated energy generation pathway is microbial rhodopsins, single-protein light-driven proton pumps that provide an auxiliary energy source and are widespread among bacteria in marine and terrestrial communities (Finkel et al. 2013). Rhodopsins require a smaller energetic investment than photosystems and a dual strategy exploiting both rhodopsins and anoxygenic phototrophy was recently shown in Tardiphaga species in a cryospheric environment (Zeng et al. 2020) and Sphingomonas AAP5 was the first physiologically characterized dual phototroph isolated from an oligotrophic alpine lake (Kopejtka et al. 2022). Rhodopsins may therefore be particularly advantageous in nutrient-scarce, high-irradiance environments. We therefore considered both AAP marker genes (*pufL, pufM, puhA*) and rhodopsin genes as complementary indicators of light-energy–assisted lifestyles across supraglacial habitats.

Given these precedents, the question arises if AAPs occur in supraglacial habitats and, if so, what is their biogeochemical potential? During summer melt on glaciers and ice sheets worldwide, the photoautotrophic biomass is partitioned across three main habitats: snow algal-dominated melting snowpacks, glacier ice algal-dominated bare ice surfaces, and cyanobacterial communities in cryoconite holes, while all habitats host diverse heterotrophic bacterial co-communities (Jaarsma et al. 2023, Feord et al. 2025, Takeuchi et al. 2025). Even though these habitats are characterized by distinct primary producers, our understanding of prokaryotic heterotrophy is diminutive. Most mechanistic insights come from studies on cryoconite ecosystems (Poniecka et al. 2018, Buda et al. 2022, Murakami et al. 2022, Zhang et al. 2023) and, to some extent, to some investigations of snow microbial communities (Krug et al. 2020, Ren et al. 2025, Takeuchi et al. 2025). However, heterotrophic processes on bare ice surfaces remain poorly characterized, and light-energy–driven heterotrophs such as AAPs may be key players in bare ice polar habitats during summer melt conditions.

As versatile heterotrophs, AAPs would likely be associated with habitats where organic matter accumulates and persists longer, such as the weathering crust on bare ice surfaces and in cryoconite holes, rather than in permanent or seasonally melting snowpacks. Beyond this hypothesized habitat selection, the seasonality of organic matter accumulation in these differing habitats is also driven by differing primary producers and this likely imposes challenges to AAPs. For example, they may have to overcome periods of starvation, when organic matter becomes scarce or inaccessible. Genomic traits that supplement energy generation during such periods by exploitation of low-energy redox couples, which are continuously available in these environments, could therefore be advantageous in supraglacial settings. Additionally, SQ degradation genes and pathways have been described in detail (Snow et al. 2021), enabling their use as markers in metagenomic datasets. During seasonal phases when phototroph biomass becomes available to heterotrophs and SQ is released through cell lysis, AAPs could contribute to SQ turnover, as reported in marine systems. Evaluating whether a similar trophic link exists in supraglacial habitats will help clarify how phototroph-derived organic matter shapes microbial community dynamics and carbon sulfur cycling on ice surfaces.

Based on these traits, we hypothesize that AAPs are not just present in supraglacial habitats, but are active and mediate critical biogeochemical reactions in supraglacial microbiomes. Here, we test this hypothesis by analysing metagenomes and metatranscriptomes from Greenland, further expanding this analysis by including over 2400 publicly available metagenomes, and ultimately characterizing the biogeochemical potential of AAPs. Our analysis emphasizes pathways relevant for sulfur cycling, exploitation of low-energy electron donors, carbon fixation, and the capacity for algal-derived SQ turnover.

## Materials and methods

### Sample collection, processing, and sequencing

Samples representing three distinct, biologically darkened supraglacial habitats were collected during the DEEP PURPLE field summer campaigns in 2019, 2020, and 2021 (Fig. 1; for details see Table S1). All campaigns took place during July and August at 61°–66° N, when field sites experience continuous or near-continuous daylight. Sampling occurred at variable times across the day as dictated by field logistics rather than on a fixed light/dark schedule and no dedicated dark-adapted sampling was performed. We therefore cannot exclude diel variation in photosynthesis gene expression of the type reported for AAPs (Tomasch et al. 2011, Kopejtka et al. 2022, 2024) and note this as a limitation when interpreting the metatranscriptome data. Samples from bare ice-darkened surfaces, dominated by glacier ice algae, as well as red, late bloom stages of snow algae that covered snow patches near the snow line or near crevasses, as well as cryoconite hole sediments, were collected and sequenced (for more details, see Halbach et al. 2022 , Perini et al. 2024, and Jaarsma et al. 2023). Samples from areas near the PROMICE weather stations MIT (on Mittivakkat glacier), the PROMICE station TAS near the Heim and Bruckner glaciers, and the PROMICE stations QAS-M and QAS-U (South—GrIS in Fig. 1), were collected. One additional set of ice core samples was collected near the QAS-U station. Sample classifications were harmonized across campaigns to reduce the diversity of habitats considered to the three chosen categories (bare ice, snow, and cryoconite hole sediments—hereafter CCO; Table S1). We applied a few additional changes in sample treatment: (i) three samples (GR20-CO6, 9, and 11) originating from ice cores spanning the transition from snow to ice (Perini et al. 2024) were reclassified here as bare ice, as the sequenced material represented the darkened ice layer representing the weathering crust surface from a previous summer that was frozen-in over winter; (ii) no distinction was made between green- and red-coloured snow: all were classified as 'snow'; (iii) four snow samples (MIT-1,2; HE1; and G20-6 detailed in Perini et al. 2024) were treated here as single samples and final metrics reported as averaged values; and (iv) this was also the case for the three technical replicates of homogenized bare ice and CCO samples (here named GR21_ice and GR21_cryoconite) analysed in Jaarsma et al. (2023).

**Figure 1:**
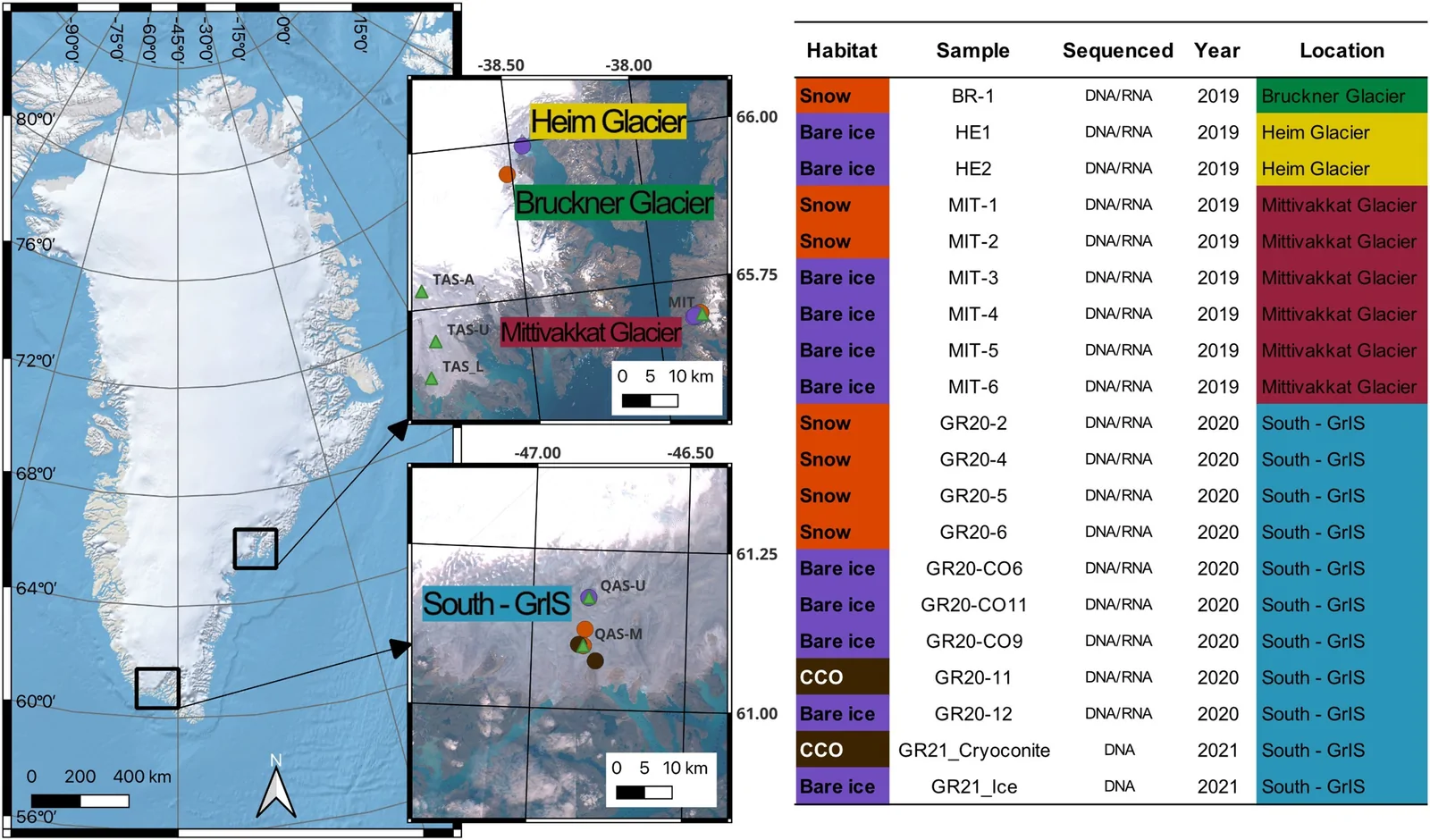
Overview of sampling locations in east and south Greenland with PROMICE stations. Samples of the three supraglacial habitats investigated shown as circles: bare ice (purple), snow (orange), and CCO (dark brown), PROMICE stations (green triangles; Andersen et al. 2016 ). Fewer samples appear on the map due to overlap of sample locations. Map was created using the QGIS package QGreenland (Moon et al. 2023), detailed maps show the Sentinel-2 multispectral satellite imagery of 2019.

Snow and ice collection was performed using field-sterilized tools, including ice axes and metal or plastic trowels (wiped with 70% ethanol and conditioned with surrounding material before sampling). For bare ice samples, the upper 2–3 cm of darkened ice was scraped off with a sterilized ice axe and collected into sterile Whirl-Pak bags. Coloured snow was similarly sampled into Whirl-Pak bags using sterilized trowels. Samples were subsequently melted at an ambient temperature (5°C–10°C) and filtered onto nitrate cellulose filters in single-use DNA filter funnels (0.22 µm, Merck Millipore^©^). Filters were folded with ethanol-cleaned forceps, placed into cryotubes, and stored in a liquid-nitrogen–conditioned cryo-shipper for transport to GFZ or Risø laboratories at Aarhus University, where they were stored at –80°C until further processing. Cryoconite hole sediment from multiple holes was collected using a polycarbonate aquarium pipette, homogenized, then subsampled into cryotubes before freezing in a liquid-nitrogen–conditioned cryo-shipper for transport.

DNA was extracted using the DNeasy PowerLyzer PowerSoil DNA Isolation Kit (Qiagen, Germany), and RNA using the RNeasy PowerSoil Total RNA Kit (Qiagen, Germany), following the manufacturer’s protocols. DNA contamination in RNA extracts was removed with the DNase Max® kit (Qiagen, Germany). Metagenomic libraries were prepared using the NEBNext® Ultra™ II FS DNA Library Prep Kit (Illumina), while metatranscriptomic libraries were constructed with the NEBNext® Ultra™ II Directional RNA Library Prep Kit for Illumina. Equimolar pools of libraries were checked on a TapeStation 4150 (D1000 DNA ScreenTape) to assess insert size distribution and detect adapter–primer dimers. Sequencing was performed on an in-house Illumina NextSeq 500 platform (300-cycle v2.5 chemistry; paired-end mode).

### Processing of metagenomes, binning, and identification of AAP_MAGs

Raw Illumina paired-end reads were adapter-trimmed and quality-filtered using FASTP v0.23.4 (Chen S 2023). Metagenomes from all samples were co-assembled with MEGAHIT v1.2.9 (Li et al. 2015) using a minimum contig length of 2500 bp, resulting in 207 653 contigs (N50 = 9080 bp). The ANVI’O suite v8 was used for organizing, comparing, and exporting genomic data for further analysis (Eren et al. 2021). A contig database was built using the program *anvi-gen-contigs-database* and 1 605 376 open reading frames (ORFs) were identified using PRODIGAL v2.6.3 (Hyatt et al. 2010). Quality-filtered reads from metagenomes and metatranscriptomes were mapped to the assembled contigs with BOWTIE2 v2.5.1 (Langmead and Salzberg 2012), SAM-files indexed and unmapped read removed with SAMTOOLS v1.17 (Danecek et al. 2021) and the resulting BAM-files sorted using the *anvi-init-bam* program in ANVI’O. BAM-files from all metagenomes and the contig database were used to create ANVI’O profiles using *anvi-profile* and, finally, two merged profiles databases from all metagenomes and metatranscriptomes were created using the *anvi-merge* program.

Contigs were binned into metagenome-assembled genomes (MAGs) using SEMIBIN2 v2.1.0 (Pan et al. 2022) and the bin collection imported into the ANVI’O database. Bins with a completion value >50 and contamination <10 based on the single copy gene collections in ANVI’O were defined as MAGs (n = 229; Table S2). Hidden Markov models (HMMs) for *pufM, pufL*, and *puhA* genes (sources of HMMs are given in the section below) were used to identify marker genes for anoxygenic phototrophy using the *anvi-run-hmms* program executing HMMER v3.4 (http://hmmer.org/). Nine MAGs showing hits for all three marker genes were classified as putative anoxygenic phototrophic MAGs and are named AAP_MAGs from here on. Those AAP_MAGs were manually refined using the interactive visualization tool of ANVI’O *anvi-refine* resulting in nine AAP_MAGs with a completion of >80% and contamination of <5% estimated by CheckM2 v1.0.1 (Chklovski et al. 2023). Taxonomic classification of MAGs was performed using the GTDB-Tk program v.2.3.2 (Chaumeil et al. 2022). Read recruitment values of metagenomes and metatranscriptomes mapped back to AAP_MAGs were generated using the *anvi-summarize* program, imported in R 4.4.0 (R Core Team 2021) and visualized using the *pheatmap* v.1.0.12 package.

### Functional annotation of ORFs

ANVIO’S built-in programs were used to add Pfam and KEGG KOfam annotations to the identified ORFs. Additional annotations were generated using METABOLIC v.4.0 (Zhou et al. 2022), HMSS2 v1.0.5 (Tanabe and Dahl 2023) for sulfur cycling, and FeGenie (Garber et al. 2020) for iron cycling genes. Briefly, a fasta file with unique gene caller IDs for every ORF in the ANVI’O database was exported and run in the respective annotation pipeline before being imported back into the database.

Several HMMs were used to identify genes of interest in our dataset. Briefly, HMMs were downloaded from TIGRFAM when available [*acsF* (TIGR02029.1), *bchE* (TIGR02026.1), *pufM* (TIGR01115.1), *pufL* (TIGR01157.1), and *puhA* (TIGR01150.2)]. To detect rhodopsin genes present in prokaryotes and *rbcL* genes we built the HMMs by downloading full length sequences (INTERPRO IPR001425 and KEGG K01601, respectively), aligning them with CLUSTAL OMEGA v1.2.4 (Sievers et al. 2011), pruning flanking regions manually with JALVIEW v2.11.4.1 (Waterhouse et al. 2009) and finally building the HMMs with the *hmmbuild* program from HMMER V3.4 (http://hmmer.org/). All downloaded or created HMMs were then used as custom HMMs within the ANVI’O suite to search ORFs using the *anvi-run-hmms* program with the -*–hmmer-program hmmsearch* flag and hits were filtered by a model-coverage threshold of 80% with the *anvi-script-filter-hmm-hits-table* program.

The python classifier AEROBOT (Flamholz et al. 2024) was used on exported fasta files of AAP_MAGs and phototrophic reference genomes to estimate oxygen requirements based on their genomic composition.

### Phylogeny of *pufLM* genes

To infer the phylogeny of *pufLM* genes of the AAP_MAGs, reference sequences were obtained from Imhoff et al. (2018). Putative *pufL* and *pufM* sequences from AAP_MAGs were retrieved from the ANVI’O contigs database using *anvi-get-sequences-for-hmm-hits*. A multiple sequence alignment was calculated from concatenated *pufLM* sequences using CLUSTAL OMEGA v1.2.4 (Sievers et al. 2011) with 100 iterations and flanking regions were manually cut from the alignment using JALVIEW v2.11.4.1 (Waterhouse et al. 2009). A tree file was generated using *IQ-TREE* v2.2.2.7 (Minh et al. 2020) with the *Model Finder Plus* algorithm for optimal model selection. The outputted tree file was annotated and visualized with iTOL (Letunic and Bork 2021).

### MAG-based comparisons

To compare functions between MAGs, gene coverage values were exported for all gene caller IDs stored in the ANVI’O contig database using the *anvi-summarize* program with *the --init-gene-coverages* flag. Gene coverages for metagenomes, metatranscriptomes of all gene calls, as well as annotations, were imported and combined in R 4.4.0 (R Core Team 2021) for all further statistical analysis and plotting using the *ggplot2* v3.5.2 package (Wickham 2016).

Random forest decision trees were built on annotated gene tables to identify functions primarily found on AAP_MAGs than other MAGs in the dataset. Trees were calculated (ntree = 2000, num.rep = 2000) with the *rfPermute* package v2.5.4 (Archer 2026). Features were ranked by their average Gini decrease across all trees, and permutation‐test *P*‐values were used to annotate significance thresholds and top functions visualized.

The completeness of selected pathways in AAP_MAGs was computed as the fraction of required marker genes detected with a coverage of >1 per MAG [100 × (number of required genes present / total required)]. Required‐gene sets were defined based on Tanabe and Dahl 2022 and 2023 for sulfur cycling genes with *soxABCDXYZ* for complete SOX gene cassettes (thiosulfate oxidation) based on Friedrich et al. (2005). All pathways definitions are summarized in Table S3. Contributions to functions in the metagenomic and metatranscriptomic signal were calculated for AAP MAGs versus other MAGs. A function was considered present on a MAG if ≥80% of the defined pathway (Table S3) was detected at a coverage of ≥1. For each passing MAG gene, coverage was normalized by gene length and proportions of the two MAG groups contributing to the pathway were calculated within each sample before averaging over the entire dataset.

### Gene-based comparison in metagenomes and metatranscriptomes

Gene coordinates on contigs were exported as a GFF3 file from the ANVI’O database and, together with the BAM files, supplied to *featureCounts* (Liao et al. 2014) to produce a matrix of short-read counts mapped to genes in the genomic datasets. Functional annotations from the ANVI’O database were exported with the *anvi-export-function* program and joined to the count matrix, along with hit tables from HMM searches, yielding one matrix with read counts per gene caller ID per sample and the corresponding annotations. This matrix was imported in R, normalized by gene length and library size, and summed across all hits per function in each sample producing depth- and length-normalized functional abundances per metagenome or metatranscriptome. Counts were then scaled by a single copy housekeeping marker (RNA_pol_L; Campbell et al. 2013) and multiplied by 100 for display as % of RNA_pol_L. Technical replicates (GR21_cryoconite1–3, GR21_Ice_1–3, MIT-1, MIT-2, HE1, and G20-6) were averaged, and averaged values for *pufL, pufM*, and *puhA* were used as a proxy for anoxygenic photosynthesis potential. Normalized levels of genes were plotted by habitat using the *ggplot2* package (Wickham 2016) and groups were statistically compared by two-sided Wilcoxon rank-sum tests using the *ggpubr* package (Kassambara 2023).

### Estimation of habitat preference using publicly available metagenomes

To assess whether the identified AAP_MAGs preferentially occur in cryogenic environments, we mapped short reads from 2451 metagenomes to the AAP_MAGs and included 30 complete genomes from fully sequenced anoxygenic phototrophs as references in this analysis. Read recruitment (genome coverage) to these genomes was used as a proxy for the presence and relative abundance of closely related populations in each metagenome. The reference genomes were retrieved from NCBI RefSeq by querying for *pufL*, yielding 99 candidates. Downloaded genomes were then dereplicated at 70% ANI with *dRep* v3.4.0 (Olm et al. 2017), resulting in 30 clusters. Dereplication thresholds vary across studies depending on the intended resolution, from ∼95%–96% ANI for species-level delineation (Olm et al. 2017) to more permissive cut-offs when the goal is to maximize phylogenetic breadth in a reference panel. We used a 70% ANI threshold here because our aim was to build a broadly representative set spanning the known diversity of anoxygenic phototrophs for habitat-preference comparisons, rather than to resolve fine-scale strain identity for read recruitment. For each generated cluster, one genome was retained as a representative.

Keyword searches for various environments where anoxygenic phototrophs occur were conducted on the SRA Explorer website (https://sra-explorer.info/) and SRA accession numbers and download links were retrieved. Metagenomes from the following environments were selected to be included in this study: glacier ice, snow, cryoconite, freshwater, microbial mats, marine sediment, lake Antarctica, salt lakes, soil biocrusts, marine Arctic, soil, hot springs, and sequenced anoxygenic phototrophic cultures as positive controls (a full list of metagenomes is provided in Table S4).

A custom bash script (see the supplementary material) iterated over accessions, screened a subset of reads with *Partie* (Torres et al. 2017) to exclude amplicon, transcriptomic, and long-read datasets and subsequently processed shotgun datasets as follows: download, quality filter with *fastp v0.23.4* (Q ≥ 5, length > 30 bp, automatic adapter trimming) (Chen S 2023), map to AAP_MAGs and reference genomes with *strobealign v0.13.0* (Sahlin 2022), convert SAM to BAM with *samtools v1.17* (Danecek et al. 2021), retain mapped reads, index, and build ANVI’O profiles with the *anvi-profile* program against a contigs database of the 39 genomes (Eren et al. 2021). Profiles were merged and a matrix showing mean coverage values of genome per metagenome generated with *the anvi-merge* and *anvi-summary* program, respectively.

To compare signals of AAP_MAGs from the GrIS with anoxygenic reference genomes across habitats, coverage data were imported in *R* v4.4.0 (R Core Team 2021) and normalized for differing numbers of genomes and metagenomes per habitat using:


\begin{eqnarray*}
{{C}_{h,g}} = \frac{{\mathop \sum \nolimits_{i \in g} \sum\nolimits_{j \in h} {C_{i,j}^\prime 1} \left( {C_{i,j}^\prime \ge T} \right)}}{{{{N}_h}{{G}_{h,g}}}}
\end{eqnarray*}

Definitions: *C_h,g_*: mean coverage per genome and detected metagenome for genome group *g* and habitat *h; C’_i,j_*: length-rescaled coverage of genome *i* in metagenome *j; T*: coverage threshold; *N_h_*: number of metagenomes in habitat *h* with ≥*T* detection; *G_h,g_*: genomes detected at least once in *h* in group *g*.

Briefly, mean coverages were normalized by dividing by each genome’s length and rescaling to the dataset mean genome size to account for variance in genome length (*C_i,j_*). Coverage values under a threshold (*T*) 0.1X were excluded to remove spurious mappings to conserved regions of genomes. For each habitat and group, normalized coverages were summed across detected genome–metagenome pairs and divided by *N_h_* and *G_h,g_*. This yields the mean coverage per genome per detected metagenome (*C_h,g_*), enabling comparison of group-specific (AAP_MAGs vs. reference genomes) signal strength across habitats. We then compared between genome groups within each habitat using a two-sided Wilcoxon rank-sum test. Prevalence values were calculated as the proportion of metagenomes within one habitat in which at least one genome in a group exceeded a coverage of 0.5X. A detailed quality check on this approach comparing mean coverage values over increasing thresholds is provided in the supplementary material (Fig. S2).

## Results

### Diverse AAPs in bare ice and CCO samples

Nine high-quality MAGs, representing 4% of the 229 MAGs recovered in this study, with an estimated completeness ranging from 83% to 100% and contamination below 4%, were identified as AAPs in samples collected over 3 years during the melt season at the GrIS from the three supraglacial habitats. These MAGs (AAP_MAG_1–9) exhibited, on average, 10-fold higher relative abundances in the bare ice and CCO habitats compared with the snow habitat (Fig. 2A). In the non-binned data, anoxygenic phototrophy (*pufL, pufM*, and *puhA*) in both metagenomes and metatranscriptomes (Fig. 2B) was also 10-fold higher in relative abundance in bare ice compared with snow samples (average of 2.6% vs. 0.2% of the total prokaryotic community, respectively). This was mirrored by the presence of rhodopsin genes in metagenomes that showed substantially higher relative abundances in bare ice compared with snow samples, but at much higher values (an average of 90% vs. 35%, respectively).

**Figure 2:**
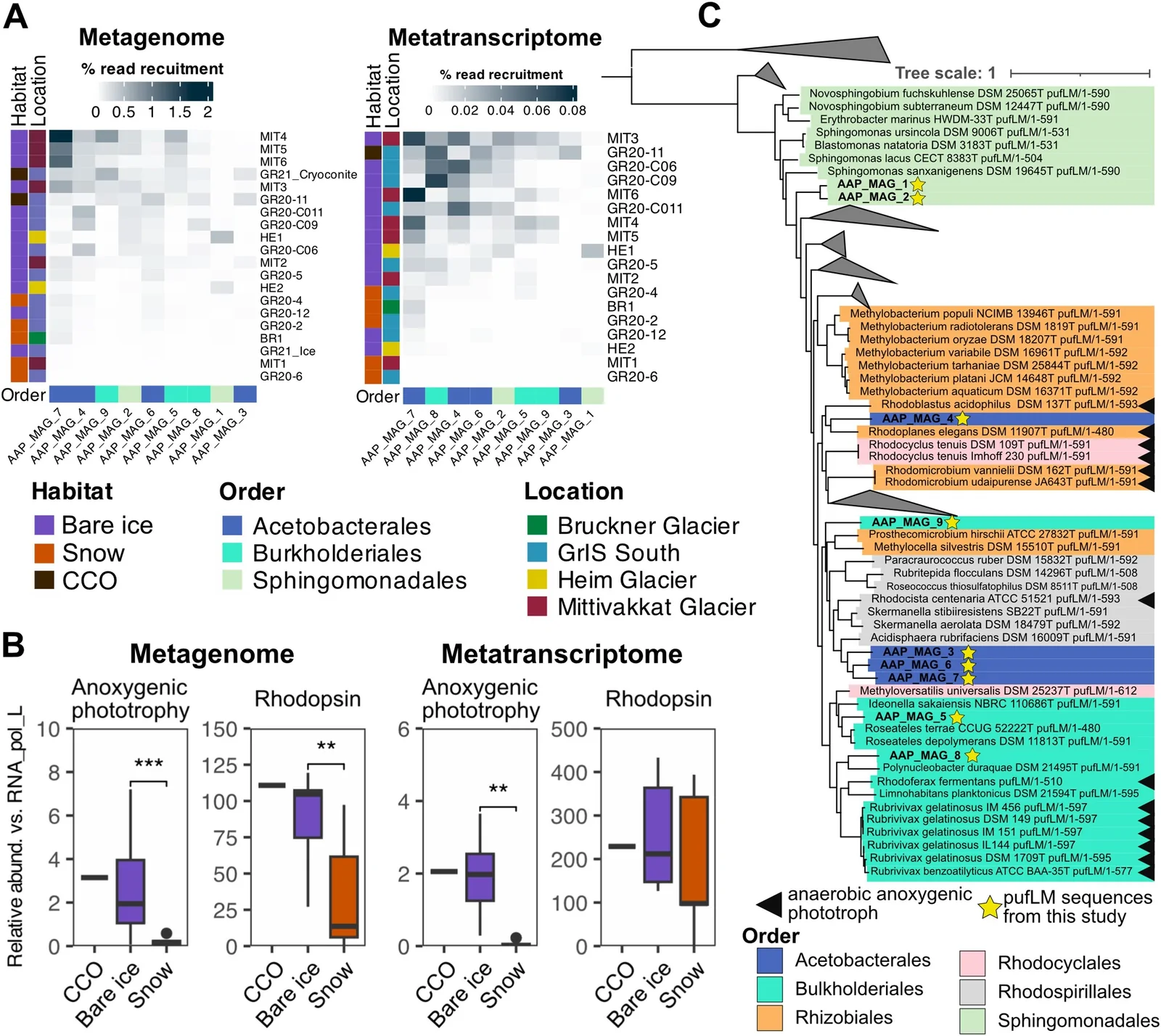
Presence, light-energy harvesting traits and phylogeny of AAP MAGs across habitats. A: Heatmap showing read recruitment to AAP_MAGs in metagenomes and metatranscriptomes, expressed as % of total reads per sample. MAGs are ordered top to bottom by their average recruitment across samples. B: Boxplots of relative abundances of anoxygenic phototrophy marker genes (pufL, pufM, puhA) and rhodopsin genes, calculated as ratios to the single-copy housekeeping gene RNA_pol_L. Wilcoxon pairwise comparisons between habitats are indicated (** p < 0.01, ^*** ^p < 0.001). Values represent community-wide relative abundance (% of single-copy RNA_pol_L signal); values >100% in metagenomes reflect general high presence of rhodopsin in the community combined with up to 4 gene copies found on single MAGs (Table S6). C: Phylogenetic placement of pufLM sequences from AAP_MAGs among reference sequences from Imhoff et al. 2018. AAP_MAG taxonomy is from GTDB Tk; reference sequence taxonomy follows Imhoff et al. 2018. An enlarged version of this tree is provided as Supplementary Fig. S3.

By contrast, no difference in rhodopsin abundances was detected in the metatranscriptomes. Comparable values to the bare ice were documented for the CCO samples, although a statistical test could not be performed due to a low sample number (N = 2, Fig. 1) from this habitat. We confirmed the observed differences by using other single copy genes to estimate the relative abundance of anoxygenic phototrophy and rhodopsin genes in the metagenomes and metatranscriptomes (Fig. S4). Overall, our results demonstrate an anoxygenic phototrophy habitat preference for bare ice and CCO compared with snow habitats.

We next assessed AAP_MAGs taxonomy and whether genome taxonomy agrees with *pufLM* phylogeny. Taxonomic classification of AAP_MAGs via comparison with the GTDB placed six MAGs in the Alphaproteobacteria (Sphingomonadales, Acetobacterales) and three MAGs in the Gammaproteobacteria (Burkholderiales) (Fig. 2A, Table S2). While GTDB-based single-copy gene taxonomy aligned with *pufLM* phylogenies for most AAP_MAGs, some discrepancies were noted (Fig. 2C). AAP_MAGs 3, 4, 6, and 7 were assigned to the order *Acetobacterales*, yet their *pufLM* sequences grouped with members of other Alphaproteobacteria (Rhizobiales and Rhodospirillales). Similarly, AAP_MAG_9 was placed within Burkholderiales (Gammaproteobacteria), but its *pufLM* sequence showed similarities to those of Rhizobiales strains (Alphaproteobacteria) in the phylogenetic tree.

The presence of *pufLM* genes alone cannot distinguish between aerobic or anaerobic lifestyles of anoxygenic phototrophs. Thus, we assessed oxygen tolerance through genomic analysis using the software Aerobot (Table S5), which, alongside the presence of the *acsF* gene (Table S6), confirmed an aerobic lifestyle for all nine AAP_MAGs. *AcsF* is a marker for aerobic bacteriochlorophyll-a synthesis (Yurkov V and Hughes 2013), as opposed to the anaerobic marker *bchE*, which was absent from these MAGs (Table S6). However, the *pufLM* sequence of AAP_MAG_4 was placed next to those of anaerobic strains in the phylogenetic tree analysis (Fig. 2C).

### Metagenome mapping to AAP_MAGs demonstrates cryospheric habitat preference

We mapped 2451 metagenomes retrieved from public repositories covering 12 environments to the AAP_MAGs to test if similar genomic signals occur in these environments (Table 1). Metagenomes from bare ice and CCO samples from this study were included as a positive control for the approach. Furthermore, 30 genomes from fully sequenced anoxygenic phototrophs representing the currently known genomic diversity of this clade were included in the analysis as a reference along with the AAP_MAGs. Datasets of shotgun-sequenced anoxygenic phototrophic cultures (AP_culture in Table 1) served as positive controls for these reference genomes. Normalized coverage per genome and metagenome (*C_h,g_*) for all environments and genome groups (AAP_MAGs and reference genomes) was calculated, where a value of 1X indicates that, on average, every base of a genome is covered by at least one read of a metagenome from this environment.

**Table 1:** Summary of normalized coverage per genome and metagenome and sample prevalence for a genome coverage of more than 50% in a metagenome across distinct environments and genome groups (AAP_MAGs and reference genomes). Number of metagenomes kept in analysis after filtering in brackets. Asterisks indicate significance level of between-groups Wilcoxon rank-sum tests (*** = *P*  < .001).

Metagenomes of distinct environments	Normalized coverage per genome and metagenome			Prevalence	
	*C_h,g_*_—AAP_MAG_	*C_h,g_*_—Ref. Genome_		AAP_MAG	Ref. Genome
**Glacier ice (83)**	**8.4X**	0.6X	***	29.6	14.1
**Cryoconite (103)**	**7.5X**	0.1X	***	59.6	5.8
Freshwater (38)	1.0X	0.3X	***	28.7	11.6
Microbial mat (42)	0.7X	0.1X	***	4.8	0.6
Marine sediment (270)	0.6X	0.2X	***	23.1	9.8
Lake Antarctica (12)	0.5X	0.4X		21.3	15.6
Salt lake (120)	0.5X	0.1X	***	9.5	3.2
Soil biocrust (90)	0.4X	0.2X	***	9.3	3.7
Marine Arctic (224)	0.3X	0.0X	***	5.9	1.2
Snow (25)	0.2X	0.1X		4.3	2.7
Soil (614)	0.2X	0.1X	***	6.5	2.7
Hotspring (182)	0.2X	0.1X	***	7.2	2
**Metagenomes included as control**					
GrIS_this_study (27)	4.0X	0.1X	***	48.6	2.6
AP culture (70)	0.0X	0.5X		0.4	1.7

Mean normalized coverage of the AAP_MAGs was highest in downloaded metagenomes of glacier ice (8.4X) and cryoconite (7.5X) samples and naturally in our GrIS (4.0X) metagenomes where they stem from. All other environments showed values of 1X (freshwater) and lower. This documents that metagenomes from glacier ice and cryoconite samples frequently contain AAP populations closely related to the AAP_MAGs at abundances sufficient to yield multi-fold genome coverage in contrast to all other environments. Reference genomes never reached the highest values of AAP_MAGs and showed mean normalized coverages of 0.6X and lower across all environments. Sequenced anoxygenic phototrophic cultures were the only data group where reference genomes showed higher values compared with AAPs, consistent with their role as positive control in this analysis.

A similar pattern was observed when we calculated ratios between metagenomes where *C_h,g_* was >0.5X versus metagenomes below this threshold, yielding the prevalence of genome groups in a specific environment. AAP_MAGs were detected in 59.6% of cryoconite and 29.6% of glacier ice metagenomes, compared with 5.8% and 14.1%, respectively, for reference genomes (Table 1). In non-cryospheric environments such as freshwater, soil, and marine sediments, the prevalence of AAP_MAGs remained higher but at lower levels, and differences to reference genomes were less pronounced.

### Functional pathways in AAP_MAGs and the broader microbiome

Random forest analysis identified thiosulfate oxidation, carbon fixation via the 3-hydroxypropionate pathway, and aerobic CO oxidation as the main features to predict an AAP_MAG compared with other MAGs in the dataset (Fig. 3A). By contrast, SQ and DMSP degradation genes were not indicative for an AAP_MAG (Fig. S1). Although iron oxidation was not found to be a predictive trait for AAP_MAGs in the random forest analysis, we included this function in further analysis as it appeared to be a widespread trait in the GrIS prokaryotic communities (Fig. 3D).

**Figure 3:**
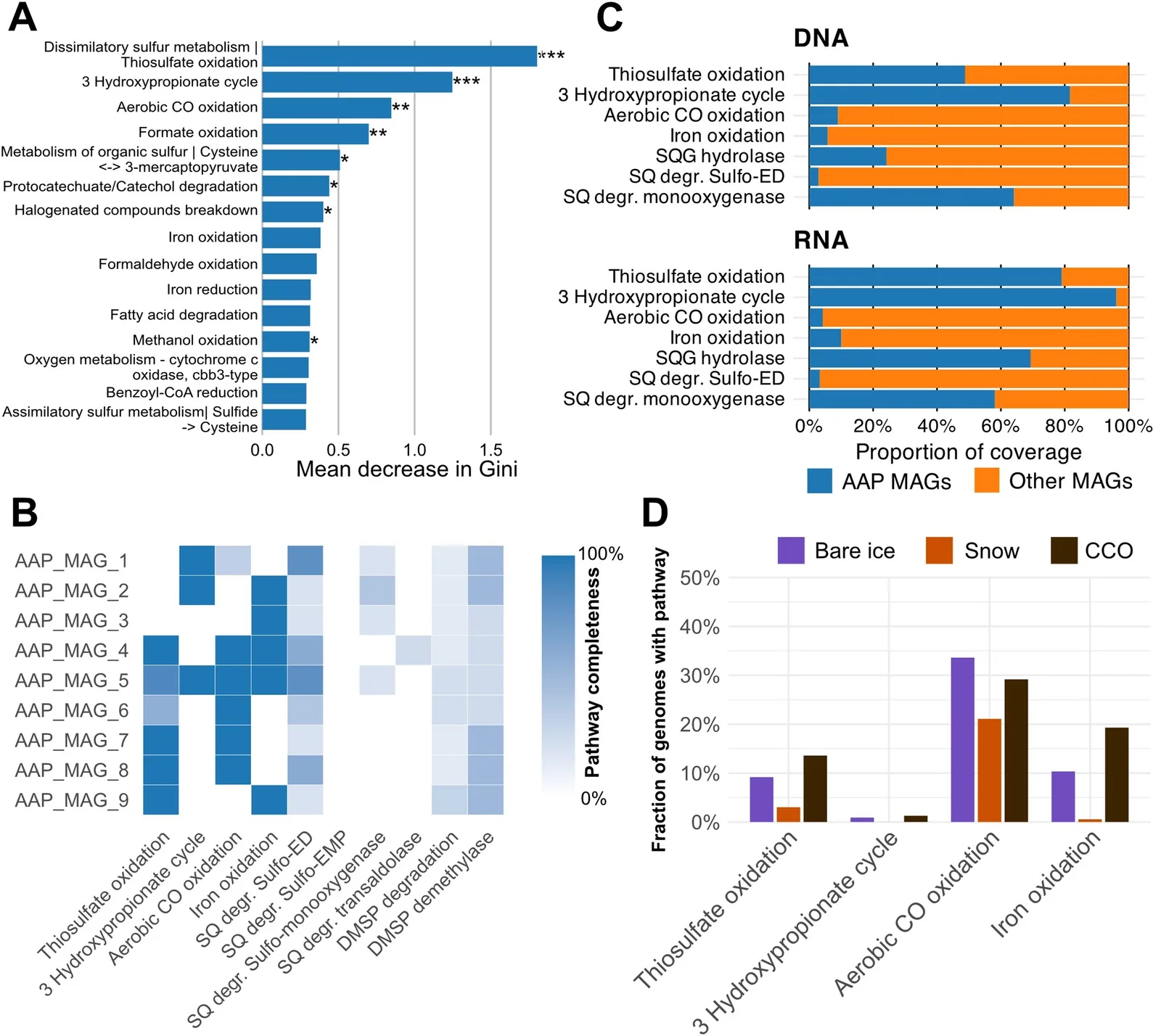
Selected pathways enriched in AAP_MAGs and their abundance across metagenomes. A: Top predictive functions distinguishing AAP_MAGs from other MAGs, ranked by Random Forest mean decrease in Gini. Bars show the average reduction in node‐impurity across all trees; larger values indicate stronger discriminative power in the model. Asterisks denote permutation‐test significance (* p < 0.05; ** p < 0.01; *** p < 0.001). B: Heatmap showing pathway completeness based on detection of required genes for selected pathways in AAP_MAGs. C: Relative contribution of AAP_MAGs versus other MAGs to each pathway's total signal in metagenomes and metatranscriptomes. Only MAGs with complete gene sets for a given pathway were included. D: Estimated fraction of genomes carrying each pathway across habitats in metagenomes. Read counts mapped to pathway genes were normalized by sequencing depth and the number of genes per pathway, then divided by counts of the single‐copy housekeeping gene RNA_pol_L to estimate genome fractions.

We calculated pathway completeness of the predictive traits for AAP_MAGs and included DMSP and SQ degradation (Fig. 3B). Such complete pathways were found for thiosulfate (4 AAP_MAGs), 3-hydroxypropionate cycle (3), aerobic CO oxidation (5), and iron oxidation (5). In addition to the 3-hydroxypropionate pathway, a gene encoding the large subunit of ribulose-1,5-bisphosphate carboxylase/oxygenase (*rbcL*, RuBisCO) was identified in AAP_MAG_3, which was corroborated by a complete M00165 KEGG module present, suggesting potential for carbon fixation via the Calvin cycle. Because RuBisCO genes are rarely reported in AAPs, the sequence was validated through a BLAST search against the NCBI NR database, confirming its identity with high similarity to *rbcL* of *Bradyrhizobium* sp. STM 3809 (94.7% identity, e-value = 0). SQ and DMSP degradation pathways were found to be absent or at lower completion levels in all AAP_MAGs.

We next compared the contribution of AAP_MAGs and all other assembled MAGs with the selected set of functional pathways in both the metagenomes and metatranscriptomes (Fig. 3C). AAP_MAGs accounted for a large share of thiosulfate oxidation potential and activity, contributing 50% of the total signal in metagenomes and 80% in metatranscriptomes. Their contribution was even higher for the 3-hydroxypropionate cycle, which was encoded almost exclusively by AAP_MAGs (>80% and >95% in DNA and RNA data, respectively). Aerobic CO oxidation and iron oxidation was found to be represented at lower levels (∼10% and 5% for DNA and RNA, respectively) by the AAP_MAGs. Sulfoquinovosidase glycosyl (SQG) hydrolases and SQ degradation via the SQ monooxygenase pathway was also a trait well represented by the AAP_MAGs (20%–60% in DNA and 50%–70% in RNA data).

Complete pathways found in AAP_MAGs (Fig. 3B) were summarized by habitat in the metagenomes (Fig. 3D) to test whether non-binned data mirror the AAP_MAG habitat pattern. Thiosulfate oxidation, iron oxidation, and carbon fixation via the 3-hydroxypropionate pathway were prominent in bare ice and CCO samples (10%–20% of total MAGs) and much lower in snow samples (0%–3%). By contrast, aerobic CO oxidation was encoded on ~25% of MAGs and found widespread in all three habitats.

### SQ and DMSP degradation potential

To assess the potential for DMSP and SQ degradation we searched for MAGs encoding complete gene sets for known degradation pathways. While DMSP degradation pathways were not found in the dataset, SQ degradation genes were identified in 22 MAGs, including four of our AAP_MAGs (Fig. 4A). Gene homologues of two distinct SQ degradation pathways were identified: the Entner-Doudoroff (Sulfo-ED) pathway, involving the *sedABCDE* genes, and the sulfoquinovose monooxygenase (SMO) pathway, involving *smoABC*. Also, the initial step of SQ degradation, cleavage of SQ from glycerol coded by the alpha-glucosidase *sftG*, was detected in six MAGs, all affiliated with the Acetobacteraceae and Sphingomonadaceae families. Of the MAGs associated with the Sulfo-ED pathway, only two (MAG_103 and MAG_131) encoded the full *sedABCDE* gene set, while nine lacked *sedD*. Within the SMO pathway, only three MAGs encoded both *smoA* and *smoB*, and no MAG contained *smoC*, suggesting incomplete pathway representation. Overall, major taxonomic groups carrying SQ degradation potential included Acetobacteraceae, Sphingomonadaceae, and Burkholderiaceae.

**Figure 4:**
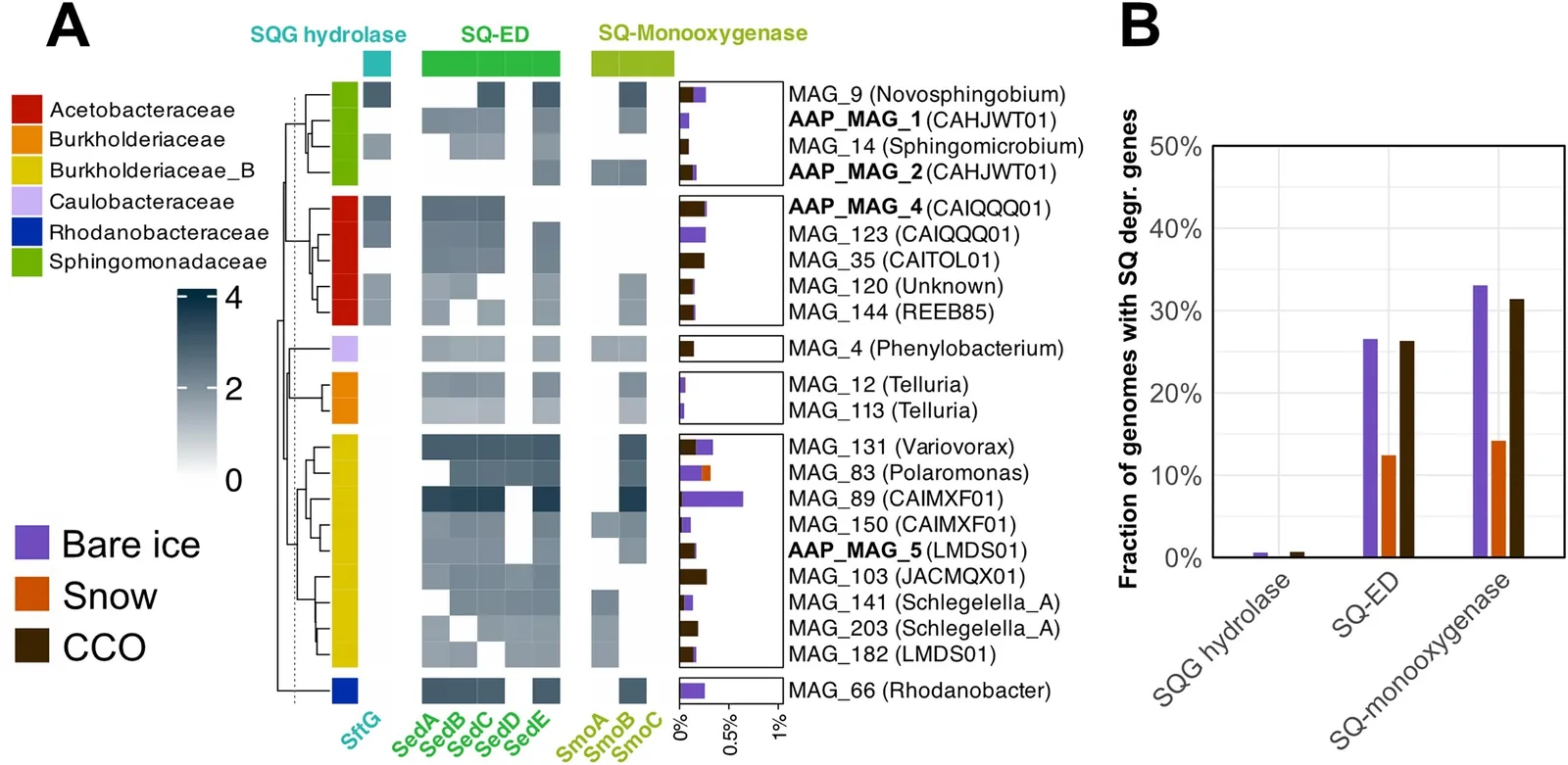
Sulfoquinovose degradation pathways detected in MAGs and their abundance across metagenomes. A: MAGs with presence of a sulfoquinovosidase gene (SQG hydrolase), at least 4 out of 5 genes for the Entner–Doudoroff-like pathway (SQ ED) or at least 2 genes in the sulfoquinovose monooxygenase pathway (SQ Monooxygenase). Heatmap shows log₁₀-transformed, sequence depth normalized gene coverage. Bar plots on the right show the relative abundance of selected MAGs across all metagenomes per habitat. B: Fraction of SQ degradation pathway genes across habitats. Read counts mapped to pathway genes were normalized by sequencing depth and divided by counts of the single‐copy housekeeping gene RNA_pol_L to estimate the fraction of genomes carrying each gene. Sample type legend as in panel A.

MAGs with SQ degradation genes were primarily associated with CCO and glacier ice surface samples (see the right-hand bars in Fig. 4A). This trend was corroborated by metagenome-level analyses that quantified the fraction of genomes containing genes from these pathways regardless of pathway completeness (Fig. 4B). In CCO and bare ice samples, 25%–33% of genomes encoded genes from the Sulfo-ED or SMO pathways, whereas these values dropped to ∼13% in snow metagenomes. The SQG hydrolase traits (*sftG*) were rare across the dataset, occurring in only ∼1% of genomes.

## Discussion

Using genome-resolved metagenomic and metatranscriptomic analyses of supraglacial habitats from Greenland, we show that AAPs are present across bare ice and CCO habitats, where they exhibit higher transcriptional activity than in snowpacks. Coupled with a broader meta-analysis, these patterns are consistent with a group of cryosphere-associated AAPs that are distinct from currently described reference lineages from other environments, thus indicating an adaptation to specialized supraglacial conditions.

### Niche specialization of AAPs on ice surfaces

Taxonomically, AAP communities belong to proteobacterial orders and the dominance of various orders has been documented for distinct habitats: marine environments are dominated by Rhodobacterales and Pseudomonadales (Ferrera et al. 2014, Stojan et al. 2024), freshwater by Acetobacterales, Rhodospirillales, Sphingomonadales, and Burkholderiales (Koblížek 2015, Kopejtka et al. 2022, Tomasch et al. 2024), and soil and phyllosphere habitats by Rhizobiales (Atamna-Ismaeel et al. 2012, Tang K et al. 2018). The AAP_MAGs identified in our samples were annotated within the Acetobacterales, Sphingomonadales, and Burkholderiales orders, consistent with AAP taxa found primarily in freshwater systems, the closest analogue to our supraglacial habitats.

Bare ice surfaces and cryoconite holes are embedded within the weathering crust, a porous ice layer that can transiently store meltwater and hydraulically link these habitats, while sustaining substantial microbial biomass within the ablation zone (Cook et al. 2016, Irvine-Fynn et al. 2021). By contrast, seasonal snowpacks that overlay the ice surface are comparatively ephemeral, accumulating during winter snowfall and typically melting out during the subsequent ablation season. These snowpacks are seeded by microbial cells and atmospheric particles delivered by wet and dry deposition from local to long-range sources (Lutz et al. 2016, Tignat-Perrier et al. 2019, Jensen et al. 2022, Keuschnig et al. 2023, McCutcheon et al. 2026). As melting progresses, deposited material is mobilized together with biomass produced by snow algal blooms. This can be transferred to the underlying bare ice, where it becomes concentrated on the surface and incorporated into the weathering crust. This seasonal redistribution contributes to the accumulation of organic and inorganic matter in longer-lived supraglacial microhabitats. These dynamics may also explain the weak AAP signal in snow, as the short residence time and repeated resetting of the snow habitat likely limits the establishment and persistence of stable heterotrophic communities before melt-out.

These habitat properties provide a mechanistic explanation for the enrichment of heterotrophs in bare ice and cryoconite hole sediment and, accordingly, for the habitat preference of AAPs observed here. We detected central carbohydrate pathways on the AAP_MAGs including glycolysis, gluconeogenesis, pentose phosphate reactions, pyruvate oxidation, and the glyoxylate shunt, together with aerobic respiratory modules such as the cytochrome bc1 complex, cytochrome-c oxidase, and ATP synthase, supporting their role as heterotrophs (Table S7).

Extending beyond Greenland, our meta-analysis showed that recruitment to GrIS AAP_MAGs is consistently strongest in weathering crust-related metagenomes (Table 1). This indicates that similar AAP communities are present on currently melting ice habitats globally, as metagenomes from Greenland, Svalbard, Asia, and also Antarctic regions mapped at high coverages to the AAP_MAGs.

AAPs thriving in cold regions under oligotrophic conditions might have phylogenetically diverged from their relatives inhabiting temperate habitats. Comparing sequences of GrIS-derived *pufLM* marker gene sequences with the currently known phylogenetic diversity indicated a complex evolutionary history (Fig. 2A and C). *PufLM* sequences from Acetobacterales-affiliated MAGs clustered with both Rhizobiales and Rhodospirillales, which aligns with the hypothesis of deep branching divergence rather than horizontal gene transfer postulated by Imhoff et al. (2018), who also found incongruent phylogenies between *16S rRNA* and *pufLM* in Rhizobiales and Rhodospirillales. In the same study, they showed that aerobic and anaerobic lifestyles of anoxygenic phototrophic cultures are spread over several taxonomic clades documenting that taxonomy alone cannot distinguish between oxygen requirements within these bacteria. For our AAP_MAGs both scenarios seemed possible as one can assume well-oxygenated bare ice surfaces and gradients of oxygen developing in cryoconite holes even reaching anoxia (Bagshaw E. A. et al. 2011, Poniecka et al. 2018, Takeuchi et al. 2025). Several approaches are now available to deduce oxygen requirements from genomic data (Jensen DB and Ussery 2013, Feldbauer et al. 2015, Podlesny et al. 2026), although it is not straightforward. For example, terminal oxidases typically found in oxygen-dependent electron transport chains can also occur in anaerobes (Lu and Imlay 2021, Berg et al. 2022). The annotation-free approach we chose targeted solely genome-derived parameters rather than detecting putative oxygen-dependent enzymes, and classified all AAP_MAGs as aerobes. This was supported by the presence of the oxygen-dependent bacteriochlorophyll biosynthesis gene (*acsF*), only found in AAPs (Yurkov V and Hughes 2013). Together with the detected transcriptomic activity of AAP_MAGs, these results further strengthen the assumption that AAPs are integral parts of supraglacial ecosystems, and counter an alternative theory where AAPs on bare ice might stem from anoxic zones in cryoconite hole sediments dispersing over the bare ice surfaces when they melt out.

Interestingly, all nine AAP_MAGs also carried rhodopsin genes in addition to the anoxygenic phototrophy operon. The dual phototrophy strategy was recently observed in *Tardiphaga* strains (Zeng et al. 2020) and *Sphingomonas* (Kopejtka et al. 2021), and likely provides a competitive advantage under the constant high light conditions of the Arctic summer. The regulation of this dual strategy may differ between taxa: in *Sphingomonas* AAP5 the photosynthesis gene cluster is repressed by glucose and likely only active under nutrient-scarce conditions, while its rhodopsin is activated specifically at low temperature and by light (Kopejtka et al. 2020, 2022). A similar temperature-dependent partitioning could be advantageous for the GrIS AAP_MAGs, as electron cycling through the bacteriochlorophyll-based reaction centre may become less efficient at low temperatures because of reduced membrane mobility of quinone and cytochrome carriers, favouring the single-protein rhodopsin pathway for capturing the abundant summer irradiance under cold conditions.

Indeed, rhodopsin genes were detected in 60% of all identified MAGs (Table S6) and showed high activity in metatranscriptomes (Fig. 2B), indicating a widespread reliance of the microbiome on this trait (Bryant and Frigaard 2006). The high abundance of rhodopsin-bearing MAGs in our samples matches values reported by Finkel et al., where 48% of the cells in 115 analysed marine and terrestrial metagenomes were found to carry rhodopsin genes (Finkel et al. 2013). However, the HMM used to identify rhodopsin genes includes not only light-driven proton and ion pumps used for energy generation, and therefore might also detect channel and sensory proteins with a photoreceptor-like function (Rozenberg et al. 2021). While an exact identification of the rhodopsin genes is beyond the scope of this study, the reported high abundance and activity points to an important role of these light-dependent functions in GrIS microbiomes.

Altogether, these findings demonstrate that AAPs occupy a niche in oxygenated bare ice and cryoconite hole sediment habitats within Greenland’s supraglacial environments. This is also corroborated by metagenomic data available in public repositories and underlines the overall importance of light-driven energy acquisition within supraglacial prokaryotic communities.

### Potential supplementing energy sources for AAPs

Supraglacial bare ice and cryoconite hole sediment habitats on the GrIS are commonly described as oligotrophic (Boetius et al. 2015), yet not limiting (Gill-Olivas et al. 2026, McCutcheon et al. 2026). These habitats receive substantial and recurring inputs of organic substrates from seasonal processes including aerosol delivery, snowpack, and ice melt, as well as the weathering crust dynamics, as outlined above. These processes accumulate biomass and exudates that can be retained and recycled within the near surface system and become available to heterotrophs (Doting et al. 2024, Peter et al. 2024, Rossel et al. 2025) or become partly exported by meltwater (Irvine-Fynn et al. 2021, Stevens et al. 2022). AAPs might be well positioned in this resource landscape because they can couple heterotrophic metabolism to light-derived energy, a strategy that can increase fitness when organic carbon and nutrients fluctuate and reach oligotrophic conditions (Ferrera et al. 2017, Kuzyk et al. 2023). Under the near continuous light of the Arctic summer, AAPs might therefore be able to offset energy limitation in periods of low organic matter supply, which potentially gives them an edge over typical heterotrophs. While AAP photoheterotrophy is consistent with their observed niche preference for ablation zone habitats, our comparative MAG analysis shows that genes for thiosulfate and putative carbon fixation pathways are enriched in AAP_MAGs relative to the broader prokaryotic community and that carbon monoxide and iron oxidation pathways are overall widespread in prokaryotic GrIS microbiomes (Fig. 3A and C). Together, these traits suggest that AAPs on the GrIS can supplement organic carbon utilization by exploiting low energy but potentially more persistent resources, as discussed below.

Thiosulfate oxidation emerged as a prominent AAP_MAG-associated function on the GrIS, with SOX-related genes and transcripts enriched in those MAGs across both metagenomic and metatranscriptomic datasets (Fig. 3). Typical electron donors that couple light energy to ATP generation in AAPs are reduced organic carbon molecules, but reduced sulfur compounds, including thiosulfate, have also been reported (Yurkov VV and Beatty 1998). On glacier surfaces, thiosulfate may originate from the abiotic oxidation of deposited pyrite-containing dust (Moses and Herman 1991). Its biological oxidation was demonstrated to exceed abiotic rates at circumneutral pH (Percak‐Dennett et al. 2017), suggesting that thiosulfate is available to AAPs under oxic conditions on the glacier surface, similar to thiosulfate-oxidizing chemolithotrophs detected beneath glaciers (Harrold et al. 2016). AAPs might therefore play critical roles in increasing the pool of bioavailable SO_4_^2-^ in these environments.

AAPs are generally regarded as photoheterotrophs that rely on organic carbon for growth, with light primarily providing auxiliary energy rather than supporting net autotrophy (Hügler and Sievert 2011, Tang et al. 2011, Hauruseu and Koblížek 2012). However, three of our AAP_MAGs encoded a complete 3-hydroxypropionate (3-HP) pathway and AAP_MAGs dominated this trait in GrIS metagenomes and metatranscriptomes overall (Fig. 3C). Genes of the 3-HP pathway were previously found to be prominent in cryoconite hole sediment and glacier forefield soil ecosystems (Zhang J et al. 2022, Chen Y et al. 2025). This carbon assimilation pathway is dependent on bicarbonate, rather than relying directly on RuBisCO-based CO_2_ fixation, as in the Calvin-Benson-Bassham (CBB) cycle, and has been discussed in the context of AAPs before (Tabita 2009, Hügler and Sievert 2011, Tomasch et al. 2011, Cai et al. 2024). In supraglacial habitats, bicarbonate availability is expected to vary with meltwater chemistry and pH, and studies on ice-lidded cryoconite systems showed that photosynthesis can raise pH while lowering pCO_2_, shifting dissolved inorganic carbon speciation towards bicarbonate and carbonate (Tranter et al. 2004, Stibal and Tranter 2007). Beyond the 3-hydroxypropionate pathway, we detected a *rbcL* gene in AAP_MAG_3 (Table S6), consistent with potential CBB cycle capacity. Genomic evidence for aerobic anoxygenic phototrophs with CBB genes has recently been reported from large-scale marine metagenomes, challenging the view that this lifestyle is strictly incompatible with carbon fixation (Graham et al. 2018). Ecologically, bicarbonate-dependent carbon assimilation via the 3-HP pathway could help AAPs persist under strong seasonality and fluctuating organic substrate supply in supraglacial habitats, particularly when meltwater chemistry favours high bicarbonate concentrations. Even modest or episodic carbon fixation by AAPs could influence supraglacial carbon budgets by reducing dependence on organic substrates.

The widespread detection of aerobic CO oxidation potential in the GrIS microbiome, including the AAP_MAGs (Fig. 3), suggests that the exploitation of this atmospheric trace gas is a critical trait for microbes in these habitats. CO oxidation has been linked to support bacterial maintenance under starvation and dormancy (King and Weber 2007, Robb and Techtmann 2018, Cordero et al. 2019), and genomic studies reported relevant genes in cryogenic settings (Franzetti et al. 2016, Ferrario et al. 2017, Ray et al. 2022, Chen Y et al. 2025). Also, CO oxidation and complementary energy acquisition with phototrophy have been connected to AAPs before (Giebel et al. 2019, Cai et al. 2024). Altogether, this supports the notion that CO oxidation is a critical trait for microbes in oligotrophic environments such as the supraglacial habitat, supplementing the metabolic profiles of AAP_MAG-like microbes reported in this study. This aligns with recent findings demonstrating the importance of atmospheric trace gas oxidation for terrestrial microbiomes (Greening and Grinter 2022).

Finally, we found that iron oxidation catalysed by the *cyc2* gene was widespread in the GrIS prokaryotic microbiome, with a habitat preference towards bare ice and CCO, consistent with the occurrence of AAPs in our dataset (Fig. 3C and D). Coupling of iron oxidation to phototrophy has only been observed in strict anoxic conditions mediated by the *pioAB* complex. This complex was not detected in our dataset and can therefore be excluded to play this role in our AAP_MAGs (Gupta et al. 2019, 2021). *Cyc2* is an outer membrane cytochrome-porin oxidizing dissolved Fe^2+^ outside the cell and is often found in neutrophilic bacteria (Keffer et al. 2021). Recent metagenomic studies in marine settings demonstrated a high diversity of *cyc2* homologues (McAllister et al. 2020, Garber et al. 2021), although its direct link to an iron oxidase function is still in question as experimental evidence is only available for a few strains so far (Castelle et al. 2008, Jeans et al. 2008). However, iron nanoparticles are ubiquitous in/on glacier and iceberg samples (Raiswell Rob 2011, Raiswell et al. 2018) and dissolved iron is also present in supraglacial snow and ice samples from the GrIS (Lutz et al. 2014, McCutcheon et al. 2021, Bradley et al. 2023 , Winkel et al. 2022). While Fe²⁺ is unstable in oxygen-rich environments, it can be generated through photoreduction of Fe³⁺ in the presence of organic matter and stabilized by complexation (Daugherty et al. 2017, Lueder et al. 2020, Kappler et al. 2021, Baker et al. 2023). Oxidation of Fe^2+^ by a *cyc2*-bearing community during snow and ice melting therefore appears plausible given the high irradiance in the polar summer and the presence of complex dissolved organic matter from the local microbiomes (Holland et al. 2019, Rossel et al. 2025).

In summary, AAPs in supraglacial habitats appear to have accumulated a range of pathways exploiting various reliably available substrates. Light, particle-derived redox couples, atmospheric trace gases, and anaplerotic carbon fixation supplement their heterotrophic lifestyle, which is in line with the overall metabolic versatility observed in this clade (Yurkov V and Csotonyi 2009, Koblížek 2015).

### SQ degradation is limited but present

Primary producers in supraglacial habitats are a potential source for SQ, which becomes available to heterotrophic communities by cell lysis (Goddard-Borger and Williams 2017, Nakajima et al. 2018). Apart from the presence of SQ in thylakoid membrane lipids (SQDG) of eukaryotic phototrophs, these organisms were also shown to remodel their cell membranes under P limitation, replacing phospholipids with non-phosphorus lipids, including SQDG (Van Mooy et al. 2006, Sebastián et al. 2016). However, recent studies demonstrate that ice melt and atmospheric deposition provide nutrients including P at levels that make macro-nutrient limitation unlikely (Gill-Olivas et al. 2026, McCutcheon et al. 2026), meaning chloroplasts released through cell lysis are likely the primary source of SQDG in these environments (Van Mooy et al. 2006, Sebastián et al. 2016, McCutcheon et al. 2021, Rossel et al. 2025, Suzuki et al. 2025).

Consistent with the potential SQ supply, homologues of SQ-degradation genes were detected in all three habitats, with approximately two-fold higher relative abundance in cryoconites and bare ice samples than in snow (Fig. 4B). We reconstructed both the SQ-Entner–Doudoroff (SQ-ED) and SQ monooxygenase pathway in GrIS MAGs, but a complete SQ-ED pathway was assembled only in two MAGs (Fig. 4A), suggesting that full SQ mineralization capacity is restricted to a small subset of taxa. Bloom dynamics of snow and glacier ice algae may further constrain actual SQ turnover. At the time of sampling, snow algal blooms may have been in later stages while ice algal blooms were ongoing or near peak intensity, potentially limiting the availability of lysed algal material to an SQ-degrading community.

Overall, our data indicate that SQ degradation potential is present in GrIS heterotrophic communities, implying that sulfolipid derived carbon and sulfur cycling similar to processes described for algal-rich ocean surface waters could occur on ice surfaces. At the same time, the limited recovery of complete pathways and the lack of a clear signal specifically linking these genes to AAP_MAGs mean our results provide only weak support for a trophic AAP-algal relationship mediated by SQ compounds, analogous to that observed in marine settings. The strong seasonal dynamics of supraglacial ecosystems may restrict SQ availability to brief periods during bloom senescence and overwinter decay. Later-stage decay, such as chytrid parasitism of ice algae, could then increase access to intracellular substrates (Perini L. et al. 2023, Perini Laura et al. 2026).

## Conclusion

Our genome-resolved analyses of GrIS metagenomes and metatranscriptomes identified AAPs as active and recurrent members of the bare ice and CCO microbiomes, with substantially weaker signals in snowpacks from eastern and southern Greenland. An extensive meta-analysis across publicly available metagenomes supports this habitat association and indicates that GrIS AAP lineages are distinct from currently represented AAP genomes, consistent with a cryosphere-associated assemblage adapted to cold, oligotrophic conditions with high seasonal variability. In addition, the high prevalence of rhodopsin genes across GrIS MAGs indicates that light-driven energy acquisition is a broadly relevant trait in these supraglacial habitats.

The metabolic pathways encoded by the AAP_MAGs support a light-assisted, heterotrophic lifestyle and suggest thiosulfate, iron, and CO oxidation, as well as anaplerotic carbon fixation as capabilities that may promote AAP persistence in nutrient-limited supraglacial habitats. While these AAPs likely contribute to the turnover of organic matter derived from primary producers within the weathering crust, where biomass and inorganic substrates are retained, we find limited support for a dominant role in sulfoquinovose degradation, as observed in marine settings.

Overall, these results broaden the current view of supraglacial microbiomes by identifying AAPs as consistent and active components of bare ice and cryoconite communities and by outlining metabolic traits that may couple light-assisted heterotrophy to sulfur cycling on glacier ice surfaces.

## Supplementary Material

fiag116_Supplemental_Files

## Data Availability

The data underlying this article are available in the Sequence Read Archive at the National Center for Biotechnology Information (https://www.ncbi.nlm.nih.gov/sra), and can be accessed with project number PRJNA1011216 for GR19 and GR20 data and PRJNA942590 for GR21 data.
