# Supplementary material for: Aerobic anoxygenic phototrophs are widespread and active members of Greenland Ice Sheet microbiomes
Source: FEMS Microbiol Ecol. 2026 Oct 15;102(11):fiag116. doi: 10.1093/femsec/fiag116 (PMC13702023; doi:10.1093/femsec/fiag116)
Supplement: Supplementary file 1 [file fiag116_supplemental_files.zip › bash_script_map_metagenomes_to_AAP_MAGs.docx]

```
#!/bin/bash
# =============================================================================
# Script: process_sra_accessions.sh
# Description:
#   This script processes SRA accessions from a metadata file (sra_explorer_metadata.tsv)
#   to identify metagenomic datasets, download raw reads, perform quality filtering,
#   map reads to a set of reference MAGs, and generate anvi'o profiles.
#
#   It uses PARTIE to classify each accession (amplicon, WGS, other) and skips
#   non-metagenomes. It also tracks already processed accessions to avoid reprocessing.
#
# Dependencies:
#   - conda environments: partie, fastp, strobealign, anvio-dev
#   - Tools: wget, samtools, esearch, efetch (NCBI E-utilities)
#   - Files required:
#       sra_explorer_metadata.tsv   : from SRA Explorer (tab-separated with cols 1=accession,9=aspera/wget link)
#       PROCESSED.txt                : empty file to record completed metagenomes
#       AMPLICON.txt                 : empty file to record amplicon datasets
#       LONG_READ_DATA.txt           : empty file to record long-read datasets (timeout from PARTIE)
#       ../processed_accessions.txt  : optional, to avoid duplicates with other scripts
#       ../MAGs_refgenomes_to_map.fasta : reference MAGs FASTA
#       ../MAGs_refgenomes_to_map.db    : anvi'o contigs database for the references
#
# Outputs:
#   - anvi_profiles/${ACCESSION}/PROFILE.db for each successfully processed metagenome
#   - metadatafiles/${ACCESSION}.metadata (SRA run info)
#   - Accession numbers appended to PROCESSED.txt, AMPLICON.txt, or LONG_READ_DATA.txt
# =============================================================================

# Initialize conda for use in script
source ~/miniconda3/etc/profile.d/conda.sh

# Create directory for metadata files (if not exists)
mkdir -p metadatafiles

# -----------------------------------------------------------------------------
# File paths for tracking progress
# -----------------------------------------------------------------------------
PROCESSED="PROCESSED.txt"                # Metagenomes that completed mapping & profiling
AMPLICON="AMPLICON.txt"                  # Accessions identified as amplicon data
LONG_READ_DATA="LONG_READ_DATA.txt"      # Accessions that caused PARTIE timeout (likely long reads)
ALL_PROCESSED="../processed_accessions.txt"  # Optional global processed list

# Set Internal Field Separator to newline only (to handle spaces in metadata lines)
IFS=$'\n'

# -----------------------------------------------------------------------------
# Main loop: process each line in the SRA Explorer metadata file
# -----------------------------------------------------------------------------
for LINE in $(cat sra_explorer_metadata.tsv); do
  # Extract accession (column 1) and download link (column 9)
  ACCESSION=$(echo "${LINE}" | awk -F '\t' '{print $1}')
  ASPERALINK=$(echo "${LINE}" | awk -F '\t' '{print $9}')

  echo "Start processing $ACCESSION $(date)"

  # ---------------------------------------------------------------------------
  # 1. Skip already processed accessions
  # ---------------------------------------------------------------------------
  # Skip if found in PROCESSED.txt (completed metagenome)
  if grep -Fxq "${ACCESSION}" "${PROCESSED}"; then
    echo "${ACCESSION} already processed - Skipping"
    continue
  fi
  # Skip if found in AMPLICON.txt (amplicon dataset)
  if grep -Fxq "${ACCESSION}" "${AMPLICON}"; then
    echo "${ACCESSION} already in AMPLICON file - Skipping"
    continue
  fi
  # Skip if found in LONG_READ_DATA.txt (long read dataset)
  if grep -Fxq "${ACCESSION}" "${LONG_READ_DATA}"; then
    echo "${ACCESSION} is long read data - Skipping"
    continue
  fi
  # Skip if found in the global processed list (optional)
  if grep -Fxq "${ACCESSION}" "${ALL_PROCESSED}"; then
    echo "${ACCESSION} processed by another script - Skipping"
    continue
  fi

  echo "${ACCESSION} has not been processed yet"

  # ---------------------------------------------------------------------------
  # 2. Use PARTIE to classify the accession (amplicon, WGS, other)
  #    PARTIE downloads 10,000 reads from the .sra file and runs a classifier.
  #    A timeout of 10 minutes is set to catch long-read datasets.
  # ---------------------------------------------------------------------------
  echo "Checking if accession ${ACCESSION} is metagenome with PARTIE"
  SRA="${ACCESSION}.sra"
  conda activate partie

  MAX_EXECUTION_TIME=$((10 * 60))   # 10 minutes in seconds
  timeout ${MAX_EXECUTION_TIME} partie.pl -nreads 10000 "${SRA}" > partie.out
  exit_code=$?

  if [ ${exit_code} -eq 124 ]; then
    # Timeout occurred → likely long-read data
    echo "PARTIE exceeded time limit - likely long read data. Exiting."
    echo "${ACCESSION}" >> "${LONG_READ_DATA}"
    rm -f "${ACCESSION}.10000.fna"   # PARTIE temporary file
    conda deactivate
    continue
  else
    echo "PARTIE executed within the time limit."
    # Run the PARTIE classification R script to generate partie_classification.csv
    Rscript ~/softwares/partie/RandomForest/PARTIE_Classification.R partie.out
  fi
  conda deactivate

  # ---------------------------------------------------------------------------
  # 3. Interpret PARTIE classification result
  # ---------------------------------------------------------------------------
  if grep -q "AMPLICON" partie_classification.csv; then
    echo "${ACCESSION} is amplicon dataset"
    echo "${ACCESSION}" >> "${AMPLICON}"
    rm -f partie_classification.csv partie.out
    continue
  fi

  if grep -q -E "WGS|OTHER" partie_classification.csv; then
    echo "${ACCESSION} is metagenome"
    # Clean up temporary files
    rm -f partie_classification.csv partie.out

    # -------------------------------------------------------------------------
    # 4. Download raw reads (using wget on the provided link)
    # -------------------------------------------------------------------------
    # Determine if dataset is paired-end (link contains "_1.fastq.gz")
    if echo "${ASPERALINK}" | grep -q "${ACCESSION}_1.fastq.gz"; then
      # -------------------- PAIRED-END DATASET --------------------
      echo "${ACCESSION} is paired end dataset"
      # Extract base URL (without _1.fastq.gz suffix)
      ASPERA_PATH=$(echo "${ASPERALINK}" | awk -F'_' '{print $1}')
      echo "FastQ URL is: ${ASPERA_PATH}"

      # Download both read files
      for i in 1 2; do
        wget "${ASPERA_PATH}_${i}.fastq.gz" ./
      done

      # -----------------------------------------------------------------------
      # 5. Quality filtering with fastp (paired-end)
      # -----------------------------------------------------------------------
      echo "Start quality filtering on paired end reads for: ${ACCESSION} $(date)"
      conda activate fastp
      fastp -i "${ACCESSION}_1.fastq.gz" \
            -I "${ACCESSION}_2.fastq.gz" \
            -o "${ACCESSION}_1_QUALITY_PASSED.fastq.gz" \
            -O "${ACCESSION}_2_QUALITY_PASSED.fastq.gz" \
            --qualified_quality_phred 5 \
            --unqualified_percent_limit 20 \
            --detect_adapter_for_pe \
            --length_required 30 \
            -w 12
      conda deactivate

      # Remove raw reads if quality-filtered files exist
      if [ -e "${ACCESSION}_2_QUALITY_PASSED.fastq.gz" ]; then
        rm -f "${ACCESSION}_1.fastq.gz" "${ACCESSION}_2.fastq.gz" \
               fastp.html fastp.json
      fi

      # -----------------------------------------------------------------------
      # 6. Map filtered reads to reference MAGs using strobealign (paired-end)
      # -----------------------------------------------------------------------
      echo "Start mapping paired end reads for: ${ACCESSION} $(date)"
      conda activate strobealign
      strobealign ../MAGs_refgenomes_to_map.fasta \
                  "${ACCESSION}_1_QUALITY_PASSED.fastq.gz" \
                  "${ACCESSION}_2_QUALITY_PASSED.fastq.gz" \
                  --threads 12 \
                  -o "${ACCESSION}.sam"
      conda deactivate

      # Remove filtered reads after mapping
      if [ -e "${ACCESSION}.sam" ]; then
        rm -f "${ACCESSION}_1_QUALITY_PASSED.fastq.gz" "${ACCESSION}_2_QUALITY_PASSED.fastq.gz"
      fi

      # -----------------------------------------------------------------------
      # 7. Convert SAM to BAM with samtools (keep only mapped reads)
      # -----------------------------------------------------------------------
      echo "Start samtools to create bam file for: ${ACCESSION} $(date)"
      conda activate strobealign  # (or any env with samtools; here we reuse strobealign)
      samtools view -F 4 \
              -bS \
              --threads 12 \
              -o "${ACCESSION}_RAW.bam" \
              "${ACCESSION}.sam"
      conda deactivate

      if [ -e "${ACCESSION}_RAW.bam" ]; then
        rm -f "${ACCESSION}.sam"
      fi

      # -----------------------------------------------------------------------
      # 8. Sort and index BAM with anvi'o (anvi-init-bam)
      # -----------------------------------------------------------------------
      echo "Start indexing paired end reads with anvio for: ${ACCESSION} $(date)"
      conda activate anvio-dev
      anvi-init-bam -T 12 \
                    -o "${ACCESSION}.bam" \
                    "${ACCESSION}_RAW.bam"

      if [ -e "${ACCESSION}.bam" ]; then
        rm -f "${ACCESSION}_RAW.bam"
      fi

      # -----------------------------------------------------------------------
      # 9. Profile BAM against the reference contigs database
      # -----------------------------------------------------------------------
      echo "Start profiling paired end reads with anvio for: ${ACCESSION} $(date)"
      anvi-profile -i "${ACCESSION}.bam" \
                   -c ../MAGs_refgenomes_to_map.db \
                   -o "anvio_profiles/${ACCESSION}" \
                   --sample-name "${ACCESSION}" \
                   -T 12

      if [ -e "anvio_profiles/${ACCESSION}/PROFILE.db" ]; then
        # Mark accession as processed and clean up intermediate files
        echo "${ACCESSION}" >> "${PROCESSED}"
        rm -f "${ACCESSION}.bam" "${ACCESSION}.bam.bai" \
              "${ACCESSION}_RAW.bam" "${ACCESSION}.sam" \
              "${ACCESSION}_1.fastq.gz" "${ACCESSION}_2.fastq.gz" \
              "${ACCESSION}_1_QUALITY_PASSED.fastq.gz" "${ACCESSION}_2_QUALITY_PASSED.fastq.gz" \
              fastp.html fastp.json

        # Fetch SRA metadata using NCBI E-utilities
        esearch -db sra -query "${ACCESSION}" | efetch -format runinfo >> "metadatafiles/${ACCESSION}.metadata"

        echo "Finished processing accession: ${ACCESSION} $(date)"
      fi

    else
      # -------------------- SINGLE-END DATASET --------------------
      echo "${ACCESSION} is single read file"
      # Download single-end file
      wget "${ASPERALINK}" ./

      # Quality filtering
      echo "Start quality filtering on single end reads for: ${ACCESSION} $(date)"
      conda activate fastp
      fastp -i "${ACCESSION}.fastq.gz" \
            -o "${ACCESSION}_QUALITY_PASSED.fastq.gz" \
            --qualified_quality_phred 5 \
            --unqualified_percent_limit 20 \
            --length_required 30 \
            -w 12
      conda deactivate

      if [ -e "${ACCESSION}_QUALITY_PASSED.fastq.gz" ]; then
        rm -f "${ACCESSION}.fastq.gz" fastp.html fastp.json
      fi

      # Mapping
      echo "Start mapping single end reads for: ${ACCESSION} $(date)"
      conda activate strobealign
      strobealign ../MAGs_refgenomes_to_map.fasta \
                  "${ACCESSION}_QUALITY_PASSED.fastq.gz" \
                  --threads 12 \
                  -o "${ACCESSION}.sam"
      conda deactivate

      if [ -e "${ACCESSION}.sam" ]; then
        rm -f "${ACCESSION}_QUALITY_PASSED.fastq.gz"
      fi

      # SAM to BAM
      echo "Start samtools to create bam file for: ${ACCESSION} $(date)"
      samtools view -F 4 \
              -bS \
              --threads 12 \
              -o "${ACCESSION}_RAW.bam" \
              "${ACCESSION}.sam"

      if [ -e "${ACCESSION}_RAW.bam" ]; then
        rm -f "${ACCESSION}.sam"
      fi

      # Sort/index BAM
      echo "Start indexing single end reads with anvio for: ${ACCESSION} $(date)"
      conda activate anvio-dev
      anvi-init-bam -T 12 \
                    -o "${ACCESSION}.bam" \
                    "${ACCESSION}_RAW.bam"

      if [ -e "${ACCESSION}.bam" ]; then
        rm -f "${ACCESSION}_RAW.bam"
      fi

      # Profile
      echo "Start profiling single end reads with anvio for: ${ACCESSION} $(date)"
      anvi-profile -i "${ACCESSION}.bam" \
                   -c ../MAGs_refgenomes_to_map.db \
                   -o "anvio_profiles/${ACCESSION}" \
                   --sample-name "${ACCESSION}" \
                   -T 12

      if [ -e "anvio_profiles/${ACCESSION}/PROFILE.db" ]; then
        echo "${ACCESSION}" >> "${PROCESSED}"
        rm -f "${ACCESSION}.bam" "${ACCESSION}.bam.bai" \
              "${ACCESSION}_RAW.bam" "${ACCESSION}.sam" \
              "${ACCESSION}_QUALITY_PASSED.fastq.gz" "${ACCESSION}.fastq.gz" \
              fastp.html fastp.json

        esearch -db sra -query "${ACCESSION}" | efetch -format runinfo >> "metadatafiles/${ACCESSION}.metadata"

        echo "Finished processing accession: ${ACCESSION} $(date)"
      fi
    fi
  fi
done

echo "End of script: all accessions in list processed."
```
