# Supplementary material for: Aerobic anoxygenic phototrophs are widespread and active members of Greenland Ice Sheet microbiomes
Source: FEMS Microbiol Ecol. 2026 Oct 15;102(11):fiag116. doi: 10.1093/femsec/fiag116 (PMC13702023; doi:10.1093/femsec/fiag116)
Supplement: Supplementary file 1 [file fiag116_supplemental_files.zip › supplementary_figures-AAPs_on_GrIS_23_7_2026.docx]

**Figure S1:** **The top 30 annotated functions with the largest impact on random forest models classifying AAP_MAGs in metagenomic and metatranscriptomic datasets**

Random forest models were constructed using annotations from A) HMSS2 and FeGenie an B) PFAM. Asterisks indicate functions with statistically significant p-values based on model permutation tests.

**Figure S2:** **Total normalized coverage per genome and metagenome for GrIS AAP_MAGs (red) and reference genomes (blue) as a function of the minimum coverage threshold (0.1X to 5X).**

Read recruitment from public metagenomes can be misinterpreted if short reads map to conserved regions shared across taxa or closely related genomes (Table 1). To assess robustness of our meta-analysis, we recalculated summary coverage statistics for both genome groups over increasing detection thresholds (0.1X to 5X in 0.01 steps) across the 13 habitats. As the threshold increases, low-coverage and potentially spurious matches are removed, which increases the mean normalized coverage among retained genome–metagenome pairs while reducing the number of detected pairs (i.e., the denominator shrinks). The resulting curves are consistent with Table 1: cryoconite, glacier ice, and the GrIS metagenomes from this study retain high AAP_MAG coverage across thresholds, whereas reference genomes remain low in these habitats. Positive controls (shotgun-sequenced anoxygenic phototrophic cultures) behave as expected, showing high coverage of reference genomes and AAP_MAG detection only at low thresholds. In most other habitats, both genome groups show similar increases with threshold, consistent with reads mapping primarily to conserved regions shared across AAP lineages rather than to the genome sets we selected for this analysis.

Overall, this analysis supports the interpretation that the AAP_MAGs described here are reliably detectable in cryogenic habitats similar to those on the GrIS, whereas signals in other habitats likely reflect related taxa and/or nonspecific mapping rather than the presence of the GrIS AAP_MAGs.

**Figure S3:** Copy of phylogenetic tree of *pufLM* tree from Figure 2C increased in size for better readability.

**Figure S4:** Boxplots of relative abundances of anoxygenic phototrophy marker genes (*pufL,* *pufM*, *puhA*) and rhodopsin genes, calculated as ratios to different bacterial single-copy housekeeping genes from Campbell et al. 2013. These comparisons consistently confirm that cryoconite holes and bare ice surfaces are habitats were anoxygenic phototrophy accumulates compared to snow habitats.
